# EDUCATION, ADULT LITERACY SKILLS, AND JOB AUTOMATION RISK IN THE OLDER US WORKFORCE

**DOI:** 10.1093/geroni/igad104.2504

**Published:** 2023-12-21

**Authors:** Donnette Narine, Takashi Yamashita, Runcie Chidebe, Phyllis Cummins, Jenna Kramer, Rita Karam

**Affiliations:** University of Maryland Baltimore, Baltimore, Maryland, United States; University of Maryland, Baltimore County, Baltimore, Maryland, United States; Miami University, Oxford, Ohio, United States; Miami University, Oxford, Ohio, United States; RAND Corporation, Arlington, Virginia, United States; RAND Corporation, Santa Monica, California, United States

## Abstract

Despite the overall benefits of technological advancement, ongoing job automation has disrupted the U.S. workforce. Of particular concern is that increasing levels of job replacement—driven by job automation—is likely to be a risk factor for economic uncertainty, both for workers and the labor market. Older workers with lower educational attainment and basic adult literacy are known to face challenges in learning new knowledge and skills, which are currently required to be competitive in a technology-driven labor market. Thus, this study aimed to examine the relationship between educational attainment, literacy proficiency, and previously published job automation risk by occupations. Using the 2012/2014 Program for International Assessment of Adult Competencies (PIAAC) data, we analyzed a nationally representative sample of middle-aged and older workers (age 45-74 years; n = 1,484). The results of the linear regression indicated that higher educational attainment (college or higher vs. less than high school: b = -14.11, p < 0.05) and greater literacy proficiency (score 0-500 points: b = -0.06, p < 0.05) were associated with lower job automation risks (0-100 % replacement in the next decades) among middle-aged and older U.S. workers, accounting for the covariates (R-squared = 0.18). In alignment with the federal Workforce Innovation and Opportunity Act, these findings offer empirical support for policies and interventions that are purposefully designed to boost the human capital (i.e., knowledge and skills) of middle-aged and older workers through adult education and literacy training to lower their job automation risk, thereby increasing their level of job security.

